# Titanium dioxide nanoparticles alleviates polystyrene nanoplastics induced growth inhibition by modulating carbon and nitrogen metabolism via melatonin signaling in maize

**DOI:** 10.1186/s12951-024-02537-x

**Published:** 2024-05-17

**Authors:** Xiaoxiao Yang, Ke Feng, Guo Wang, Shifang Zhang, Juan Zhao, Xiangyang Yuan, Jianhong Ren

**Affiliations:** 1 College of Life Sciences, Shanxi Agricultural University, Taigu, 030800 Shanxi China; 2 State Key Laboratory of Soil Erosion and Dryland Farming on the Loess Plateau, College of Life Sciences, Northwest A&F University, Yangling, 712100 Shaanxi China; 3 College of Agriculture, Shanxi Agricultural University, Taigu, 030800 Shanxi China

**Keywords:** Nanoplastics, Nano-TiO_2_, Carbon/nitrogen, Antioxidant, Melatonin

## Abstract

**Background:**

Nanoplastics, are emerging pollutants, present a potential hazard to food security and human health. Titanium dioxide nanoparticles (Nano-TiO_2_), serving as nano-fertilizer in agriculture, may be important in alleviating polystyrene nanoplastics (PSNPs) toxicity.

**Results:**

Here, we performed transcriptomic, metabolomic and physiological analyzes to identify the role of Nano-TiO_2_ in regulating the metabolic processes in PSNPs-stressed maize seedlings (*Zea mays* L.). The growth inhibition by PSNPs stress was partially relieved by Nano-TiO_2_. Furthermore, when considering the outcomes obtained from RNA-seq, enzyme activity, and metabolite content analyses, it becomes evident that Nano-TiO_2_ significantly enhance carbon and nitrogen metabolism levels in plants. In comparison to plants that were not subjected to Nano-TiO_2_, plants exposed to Nano-TiO_2_ exhibited enhanced capabilities in maintaining higher rates of photosynthesis, sucrose synthesis, nitrogen assimilation, and protein synthesis under stressful conditions. Meanwhile, Nano-TiO_2_ alleviated the oxidative damage by modulating the antioxidant systems. Interestingly, we also found that Nano-TiO_2_ significantly enhanced the endogenous melatonin levels in maize seedlings. *P*-chlorophenylalanine (*p*-CPA, a melatonin synthesis inhibitor) declined Nano-TiO_2_-induced PSNPs tolerance.

**Conclusions:**

Taken together, our data show that melatonin is involved in Nano-TiO_2_-induced growth promotion in maize through the regulation of carbon and nitrogen metabolism.

**Graphical Abstract:**

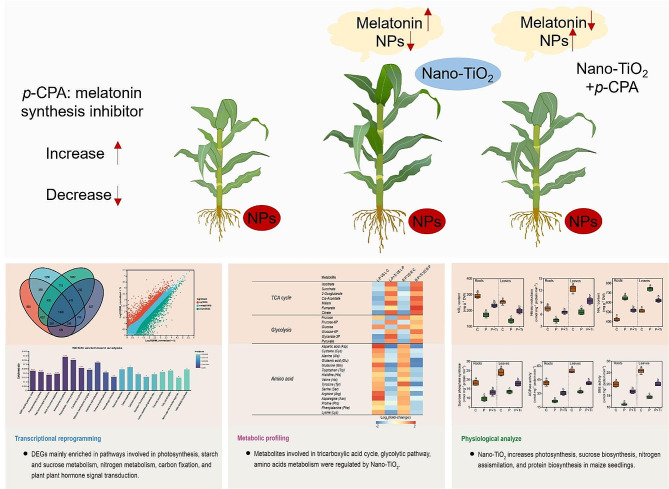

**Supplementary Information:**

The online version contains supplementary material available at 10.1186/s12951-024-02537-x.

## Introduction

Plastic pollution is a significant contemporary environmental issue. Global statistics indicate that an annual production of 0.19-83 million tons of plastic waste occurs, encompassing prevalent variants like polystyrene (PS), polyvinyl chloride (PVC), and polyethylene (PE) [1]. Upon introduction into the environment, plastic undergoes a gradual process of degradation facilitated by ultraviolet radiation, oxidation, mechanical abrasion, and other factors [2]. This degradation results in the formation of minute plastic fragments, including microplastics (MPs, 1 μm-5 mm) and nano-plastics (NPs, 1–100 nm) [3]. MPs and NPs exhibit extensive distribution within farmland, primarily originating from long-term residue of agricultural mulch, sewage irrigation, sludge application in agriculture, organic fertilizer utilization, and atmospheric deposition [4]. Research has demonstrated that MPs can inhibit plant growth through mechanisms such as decreased photosynthesis, hindered mineral element uptake, and disrupted metabolic balance [5, 6]. In contrast to MPs, NPs possess smaller dimensions and a greater specific surface area, rendering them more readily assimilated by organisms. The accumulation of NPs within plants may ultimately result in their entry into the human body via the food chain, thereby potentially posing a significant health hazard [7].

The potential applications of nanotechnology in agricultural production, including the enhancement of nutrient utilization efficiency and alleviation of abiotic stresses, have garnered significant attention from scientists [8]. Nanomaterials possess distinct characteristics such as small particle size, large specific surface area, high surface energy, a significant proportion of surface atoms, and three unique effects, namely surface effect, small size effect, and macroscopic quantum tunneling effect [9, 10]. Recent studies have demonstrated the promising prospects of Nano-TiO_2_ in alleviating environmental stresses [11, 12]. The mechanisms through which Nano-TiO_2_ increase plant stress resistance encompass the augmentation of antioxidant capacity, enhancement of photosynthetic efficiency, and improvement of element uptake [13–15]. Nevertheless, the potential of Nano-TiO_2_ to alleviate the inhibitory impact of NPs stress on crop growth, as well as the underlying mechanism, remains unclear. This study aims to address this knowledge gap.

Carbon (C) and nitrogen (N) are pivotal constituents within plants, and their respective metabolisms hold paramount significance in plant physiology. The interplay between C and N metabolism is highly intricate, as they exhibit interdependence and mutual constraint [16, 17]. On one hand, N metabolism necessitates the energy generated by C metabolism, as well as the reducing agents produced through photosynthesis, alongside the C skeleton derived from the tricarboxylic acid cycle (TCA). On the other hand, C metabolism relies on N metabolism to furnish enzymes and light pigments, with both processes relying on shared reducing power, ATP, and C skeleton [18]. The degree of coordination between C and N metabolism has significant implications for crop growth, development, yield formation, and quality [19–21]. Consequently, it is crucial to ensure the maintenance of relative stability in C and N metabolism when crops are exposed to NPs stress.

Melatonin, a multifunctional regulatory molecule found in a wide range of living organisms, including prokaryotes and eukaryotes. In animals, melatonin plays a crucial role in regulating various physiological processes, including circadian rhythm, seasonal reproductive development, immunity enhancement, and sleep improvement [22]. The discovery of melatonin in plants during the early 1990s has garnered significant attention from researchers worldwide [23]. Due to its structural similarities and functional overlap with auxin, as well as its ability to freely move within tissues, scavenge free radicals, and respond to light, melatonin is increasingly recognized as a novel plant growth regulator [24]. Research has demonstrated that melatonin possesses the ability to retard the aging process, augment photosynthesis, regulate photoperiodicity, and influence seed germination and root morphogenesis. Moreover, endogenously induced or exogenously applied melatonin has been shown to enhance plant tolerance to various abiotic stresses [25, 26]. Previous studies have indicated that melatonin can mitigate the deleterious impacts of environmental stresses, such as drought and salinity, on plant growth [27, 28]. Furthermore, exogenous application of melatonin has been shown to enhance the photosynthetic efficiency, modulate carbon and nitrogen metabolism, stimulate the accumulation of amino acids and their derivatives, and thus alleviate stress induced growth inhibition and yield reduction in plants [16, 29].

Plants have evolved diverse defense mechanisms, encompassing DNA methylation modification, histone post-translational modification, transcription and metabolic reprogramming, as well as the synthesis of secondary metabolites, to effectively cope with environmental fluctuations and withstand external stressors [30, 31]. The response mechanisms to NPs toxicity have been investigated in several plant species, such as rice and soybean, through transcriptome profiling. Numerous genes associated with mitogen-activated protein kinase (MAPK), redox reactions, and membrane protein transport regulated by NPs stress have been documented [7, 32]. Furthermore, downstream metabolites and enzymes associated with these genes significantly contribute to the plant’s response to abiotic stress [33, 34]. Consequently, the comprehensive integration of transcriptome, metabolome, and physiological approaches is essential for a comprehensive understanding of the regulatory effects of Nano-TiO_2_ on crop growth and metabolism under NPs stress.

Maize (*Zea mays* L.) is a significant staple and fodder crop, possessing substantial production capabilities and considerable economic advantages, while also serving as a versatile resource for diverse industrial applications [35]. In this study, PSNP_S_ was selected as a representative of NPs due to its prevalence in agricultural soils [36]. Nano-TiO_2_ has been recognized for its significant contribution to enhancing plant tolerance against abiotic stress. However, the potential of Nano-TiO_2_ in mitigating phytotoxicity caused by NPs remains unverified. The process of biomass accumulation, which involves the intricate interplay of various metabolic pathways, particularly the interaction between carbon and nitrogen metabolism, serves as a direct manifestation of plant growth [37]. Considering the important contribution of C and N metabolism and melatonin to plant growth, we hypothesized that Nano-TiO_2_ might enhance the carbon and nitrogen metabolism of maize plants under PSNPs stress, thereby reducing the inhibitory effect of PSNPs toxicity on maize growth, and melatonin might be involved in the regulation of this process. Furthermore, we also examined the impact of Nano-TiO_2_ on alleviating oxidative stress in maize plants.

## Materials and methods

### Plant growth and experimental design

The maize variety used in this study was Zhengdan 958. Nano-TiO_2_ (10 nm, 99.99%) were purchased from US Research Nanomaterials located in Houston, TX, USA. The PSNPs (50 nm) were purchased from Zhongkeleiming Technology Co., Ltd. (Beijing, China). The experiment was conducted within an artificial climate chamber, maintaining a photosynthetic photon flux density (PPFD) of 600 µmol m^− 2^ s^− 1^, a relative humidity range of 45–55%, and a light/dark cycle of 14/10 hours. Prior to experimentation, the seeds underwent a disinfection process involving immersion in a 1% sodium hypochlorite solution for a duration of 10 min, followed by three rinses with distilled water. The sterilized seeds were subsequently placed on filter paper and incubated at a temperature of 25 °C for a duration of 3 days in a dark environment to stimulate germination. Maize seedlings exhibiting consistent growth were then transplanted into a plastic container containing a 1/2 Hoagland solution (pH = 5.8). Based on our preliminary experimental findings, the optimal concentration of Nano-TiO_2_ was determined to be 50 mg/L. Following a period of 14 days, the seedlings were subjected to treatment with either 50 mg/L or 0 mg/L of Nano-TiO_2_ through foliar spraying. Subsequently, after a 12-hour pretreatment with Nano-TiO_2_, the nutrient solution was supplemented with 20 mg/L PSNPs to induce NPs stress. In the experiment, four treatment groups were established: the control group (C), the Nano-TiO_2_ group (Ti, with a concentration of 50 mg/L Nano-TiO_2_), the PSNPs treatment group (P, with a concentration of 20 mg/L PSNPs), and the PSNPs + Nano-TiO_2_ treatment group (P + Ti, with a concentration of 20 mg/L PSNPs + 50 mg/L Nano-TiO_2_). Each treatment group consisted of 10 replicates, with 12 seedlings per replicate. Following a 7-day treatment period, the roots and leaves from each treatment group were collected and stored at -80 °C.

### Measurement of PS concentration and plant growth parameters

The content of PSNPs in all samples was determined following the methodology outlined by Li et al. [38]. Freeze-dried leaves and roots were digested using a 25% solution of tetramethylammonium hydroxide. The resulting solution was then flocculated with anhydrous ethanol, followed by centrifugation. Subsequently, the solution was extracted using dichloromethane. The extracted substance was then analyzed using the Py-GC/MS. Following a seven-day treatment, the roots and ground parts were gathered and subjected to a drying process at a temperature of 70 °C for a duration of 72 h. Subsequently, the dry weight was determined. The root vigor was assessed using the procedure outlined by Qi et al. [39]. A fresh sample weighing 0.1 g was combined with 5 mL of 0.4% TTC solution and 5 mL of 1/15 mol/L PBS. The mixture was thoroughly mixed and incubated in a constant temperature chamber at 37℃ in the absence of light for a duration of 2 h, resulting in the appearance of red coloration in the root tip segments. Subsequently, 2 mL of 1 mol/L H_2_SO_4_ solution was added to terminate the reaction. The absorption value was then measured using the UV-2550 spectrophotometer (Shimadzu, Japan) at a wavelength of 485 nm. The determination was conducted in triplicate.

### Transcriptome sequencing and data analysis

Total RNA was isolated from the leaves and roots of both treated and control seedlings using TRIzol reagent (Invitrogen) following the guidelines provided by the manufacturer. Subsequently, PCR amplification was conducted, and high-throughput sequencing was conducted on the Novogene Technology Co., Ltd. HiSeq 2500 Illumina sequencing platform. DEGseq2 was employed to detect the Differentially Expressed Genes (DEGs; qvalue < 0.05, |log_2_FoldChange| > 1) between the control and treatment groups. The Kyoto Encyclopedia of Genes and Genomes (KEGG) enrichment analysis of DEGs was conducted using the KOBAS software. To verify the credibility of the RNA-Seq data, a random selection of eight DEGs was made and their expression patterns were examined through qRT-PCR, with the *ZmUbi-2* gene serving as the internal reference. The primer sequences employed in this analysis can be found in Table Supplemental Table S1. The relative gene expression level was determined using the comparative Ct method. Each treatment included three biological replicates.

### Metabolite extraction and identification

Frozen leaf or root samples weighing 0.8 g were pulverized into powder using liquid nitrogen under freezing conditions. Subsequently, 1.2 mL of a 70% methanol solution was added to facilitate extraction. The resulting extract was refrigerated at a temperature of 4 °C for an overnight period. Following this, centrifugation was carried out at a speed of 12,000 r/min for a duration of 10 min, and filtration was performed using a microporous filter membrane. The resulting supernatant was utilized for UPLC-MS analysis. The quantification of metabolites was conducted using the multi-reaction mode of a triple quadrupole mass spectrometry instrument. The samples underwent multivariate statistical analysis, which included principal component analysis. To further elucidate the distinctions between groups, the metabolome data was analyzed using the orthogonal partial least squares discriminant analysis (OPLS-DA) model. The screening criteria for identifying differential metabolites were set at *p* < 0.05, Log_2_Foldchange > 1, and VIP > 1. Each treatment included six biological replicates.

### Assays of chlorophyll content and photosynthetic parameters

Approximately 0.2 g of leaf samples, previously frozen at -80℃, were taken and mixed with 5 mL of 80% acetone. The resulting mixture was homogenized through grinding, followed by centrifugation at 5000 g and 4℃ for a period of 10 min. The light absorption value was measured using the UV-2550 spectrophotometer (Shimadzu, Japan) at wavelengths of 645 nm, 652 nm, and 663 nm. Following a stress treatment period of 7 days, the photosynthetic rate of maize plants was assessed using the LI-6800 portable photosynthesis analysis system (LI-COR, USA). The measurement was conducted using a 2 × 3 cm red and blue light source leaf chamber, with a light intensity of 600 µmol m^− 2^ s^− 1^. The flow rate was set at 500 µmol s^− 1^, and the relative humidity was maintained at 50%. The entire measurement process took place between 9:00 AM and 11:00 AM. The chlorophyll fluorescence parameters were determined using the PAM2500 instrument (Walz, Germany) following a 30-minute period of dark adaptation.

### Analysis of activities of enzymes involved in carbon/nitrogen metabolism

The activity of ribulose bisphosphate carboxylase (Rubisco) was assessed using the methodology outlined by Bota et al. [40]. Frozen samples weighing 0.1 g were homogenized in a 100 mM bicine-NaOH buffer (pH = 7.8) containing EDTA (1 mM), DTT (5 mM), MgCl_2_ (5 mM), and phenylmethyl sulfonyl fluoride (PMSF, 1 mM). The resulting homogenates were centrifuged at 15,000 × g and 4 °C for 5 min. The enzyme solution is then added to the mixture, which consists of Tris-HCl (200 mM, pH = 8.5), 1 mM RuBP, 10 mM NaHCO_3_, 5 mM MgCl_2_, 0.1 mM DTT, 1 mM ATP, phosphoglycerate kinase (5 Units), glyceraldehyde 3-phosphate dehydrogenase (5 Units), and 0.2 mM NADH. Spectrophotometer (UV-2550, Shimadzu, Japan) recorded absorption values at 340 nm. The activity of ADP glucose pyrophosphorylase (AGPase) was assessed following the protocol outlined by Prathap et al. [41]. The activity of amylase (AMY) was determined using the methodology described by Reguera et al. [42]. The activity of sucrose phosphate synthase (SPS) was determined based on the research conducted by Ali et al. [43], while the activity of sucrose synthase (SuSy) and invertase (INV) was determined following the procedures established by Prathap et al. [41]. The activities of citrate synthase (CS) and phosphoenolpyruvate carboxylase (PEPC) were analyzed as detailed in Zhao et al. [18].

The method described by Gangwar et al. [44] was used to determine the activity of nitrate reductase (NR), with some modifications. Frozen samples weighing 0.1 g were ground into powder under liquid nitrogen freezing conditions. An extract containing 50 mM KH_2_PO_4_-KOH (pH = 7.5), 2 mM EDTA, 2 mM dithiositol, and 1% polyvinylpyrrolidone was added to the powdered samples. The extracts were then homogenized and centrifuged for 20 min at 4 °C and 20,000×g. To initiate the reaction, 700 µL of reaction buffers (50 mM KH_2_PO_4_-KOH [pH = 7.5], 10 mM KNO_3_, and 0.1 mM NADH) were added to 100 µL of total soluble protein. The samples underwent incubation at a temperature of 28 °C for a duration of 15 min. Subsequently, the reaction was halted and the concentration of nitrite (NO_2_^−^) was assessed. The enzymatic activities of glutamine synthetase (GS) and glutamate synthetase (GOGAT) were determined utilizing the Khan et al. [45] methodology, whereas the activity of glutamate dehydrogenase (GDH) was determined following the procedure outlined by Zhao et al. [18]. The activity of protease was assessed using the methodology outlined by Reguera et al. [42]. Biological replicates were measured four times for each treatment.

### Measurement of contents of carbohydrates and nitrogen metabolites

The contents of sucrose, fructose, and glucose were determined following the methodology outlined by Liu et al. [46]. Frozen samples weighing 0.1 g were pulverized into a powder and mixed with 1 ml of 80% ethanol. The resulting extract was subjected to centrifugation at 12,000 rpm for 15 min, and the resulting samples were dissolved in 80% acetonitrile for quantitative analysis using HPLC (Agilent 1260, Santa Clara, USA). Starch content was determined using the Starch Assay Kit (STA20, Sigma-Aldrich, St. Louis, USA) in accordance with the procedure described by Dong et al. [47].

The quantification of nitrate (NO_3_^−^) was conducted using HPLC (Agilent 1260) following the methodology established by Xu et al. [48]. The determination of ammonia (NH_4_^+^) content was performed based on the procedure outlined by Oliveira et al. [49]. The assessment of free amino acid content was carried out according to the methodology described by Ghani et al. [50]. The determination of soluble protein content was conducted by Sapre et al. [51]. Frozen samples weighing 0.1 g were pulverized under freezing conditions using liquid nitrogen, and a phosphate buffer containing 2% (w/v) polyvinylpyrrolidone (PVP) and 1 mM EDTA-Na_2_ was introduced. Following homogenization, the sample underwent centrifugation for 15 min at a temperature of 4 °C and a speed of 11,000×g. The absorbance value of the sample was measured using the UV-2550 spectrophotometer (Shimadzu, Japan) at a wavelength of 595 nm, and the protein concentration was determined by employing the standard curve of bovine serum albumin (BSA) (Sigma-Aldrich, USA). All measurements were performed in triplicate.

### Assays of reactive oxygen species and antioxidant activity

The determination of superoxide anion radical (O_2_^•−^) content followed the protocol described by Altaf et al. [52]. A frozen root sample weighing 0.2 g was ground into a powder and combined with 2 mL of 50 mM phosphate buffer at pH 7.8. After homogenization, the sample was subjected to centrifugation at 4 °C and 12,000×g for 20 min. The resulting supernatant (0.5 mL) was mixed with 0.1 mL of hydroxylamine hydrochloride (10 mM) and 0.5 mL of phosphate buffer (50 mM, pH 7.8), and incubated at room temperature for 30 min. Subsequently, 1 mL of sulfanilamide (17 mM) and 1 mL of naphthylamine (7 mM) were added to the mixture, followed by another incubation at room temperature for 30 min. The measurement of the absorption value was conducted using the UV-2550 spectrophotometer (Shimadzu, Japan) at a wavelength of 530 nm. The determination of O_2_^•−^ content was accomplished by employing the standard curve of NaNO_2_ (Sigma-Aldrich, USA). The quantification of hydrogen peroxide (H_2_O_2_) and malondialdehyde (MDA) was carried out following the procedure outlined by Lai et al. [53]. The determination of superoxide dismutase (SOD) activity followed the methodology outlined by Thabet et al. [54]. Catalase (CAT) activity was determined in accordance with the method described by Altaf et al. [42], while peroxidase (POD) activity was determined following the approach detailed by Gao et al. [55]. The activities of ascorbate peroxidase (APX) and glutathione reductase (GR) were determined using the methodology described by Thabet et al. [54]. The quantification of ascorbic acid (AsA) content was analyzed based on the procedure described by Tan et al. [56].

### Effect of exogenous application of melatonin and *p*-chlorophenylalanine (*p*-CPA) on PSNPs tolerance

To enhance comprehension of the correlation between melatonin and the benefit triggered by Nano-TiO_2_, we performed an experiment involving the exogenous administration of melatonin or a melatonin synthesis inhibitor (*p*-CPA). Uniform seedlings (same as above) were subjected to five treatments: a control group without Nano-TiO_2_ (C), a group treated with 20 mg/L PSNPs (P), a group treated with 20 mg/L PSNPs (P) + 50 mg/L Nano-TiO_2_ (P + Ti), a group treated with 20 mg/L PSNPs (P) + 10 µM melatonin (P + M), and a group treated with 20 mg/L PSNPs (P) + 50 mg/L Nano-TiO_2_ + 30 µM *p*-CPA (P + Ti + CPA). After a seven-day treatment, the roots and leaves from each treatment were collected.

### Measurement of endogenous melatonin content

In accordance with the methodology outlined by Chen et al. [57], the endogenous melatonin levels were quantified using HPLC (Agilent 1260, Agilent Technologies). Fresh plant samples weighing 0.5 g were pulverized into a fine powder under cryogenic conditions using liquid nitrogen, followed by the addition of 5 mL of methanol. Subsequently, the samples were subjected to centrifugation at 10,000×g at 4 °C for 30 min, after which the methanol was removed through evaporation using a nitrogen blower. The sample was subsequently dissolved in a 0.2 mL solution of 0.1 M Na_2_HPO_4_: acetonitrile mixture (65:35) and filtered using a 0.22 μm filter membrane. A 5 µL aliquot of the sample was then injected into a C18 column maintained at 30 °C. The elution of samples was carried out at a flow rate of 0.5 mL/min and monitored at 220 nm. The endogenous melatonin content was determined by referencing a melatonin (Sigma-Aldrich, USA) standard curve.

### Statistical analysis

Data was analyzed by one-way ANOVA followed by Duncan’s tests (SPSS, version 22). Different letters indicate statistically significant differences. Values are presented as mean ± SD (*n* = 4).

## Results

### Nano-TiO_2_ alleviates PSNPs-induced growth inhibition in maize plants

**Fig. 1:**
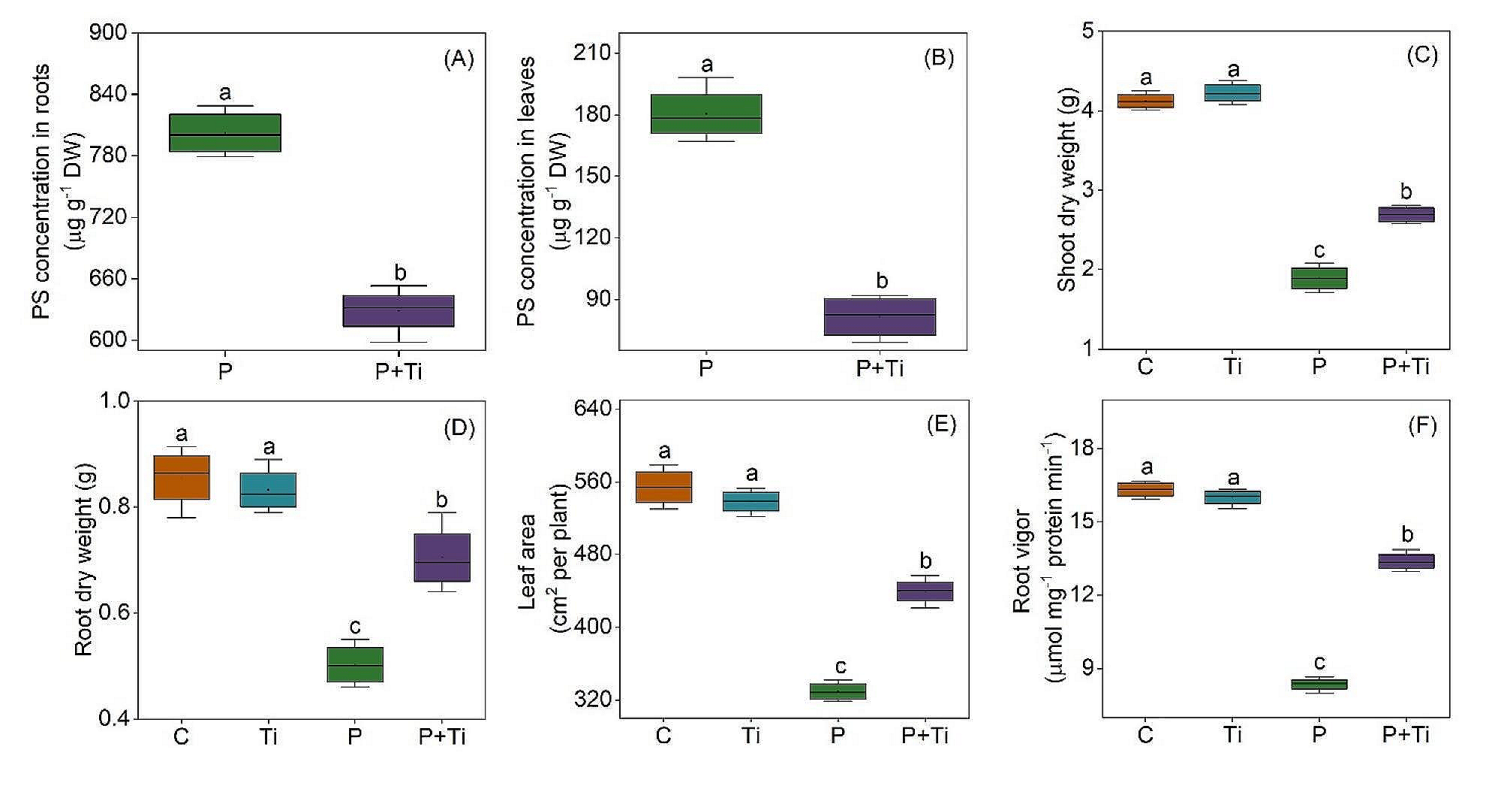
Effects of Nano-TiO_2_ on PS concentration and growth characteristics in PSNPs-stressed maize plants. (**A, B**) root/leaf PS concentration; (**C, D**) shoot/root dry weight; (**E**) leaf area; (**F**) root vigor. C: Control; Ti: Nano-TiO_2_; P: PSNPs; P + Ti: PSNPs + Nano-TiO_2_. Different letters indicate statistically significant differences (*n* = 4, *P* < 0.05)

The exposure to PSNPs stress led to a notable increase in the PS content found in the leaves and roots of maize plants. Conversely, the application of Nano-TiO_2_ treatment resulted in a dramatic reduction in PS content in the leaves and roots (Fig. 1A and B). When the plants were grown under normal conditions, the application of Nano-TiO_2_ did not have a significant impact on the dry weight of the above-ground parts of the plants. However, under PSNPs stress, there was a notable decrease of 54.1% in the above-ground dry weight. Following treatment with Nano-TiO_2_, this reduction was reduced to only 34.7%. Furthermore, the application of Nano-TiO_2_ significantly increased the dry weight of the roots (Fig. 1C and D). Similarly, the application of Nano-TiO_2_ NPs demonstrated a substantial enhancement in both leaf area and root activity (Fig. 1E and F). In light of these observations, it can be concluded that Nano-TiO_2_ possess the potential to effectively mitigate the suppressive impact of PSNPs stress on the growth of maize.

### Nano-TiO_2_ induced transcriptional reprogramming

**Fig. 2:**
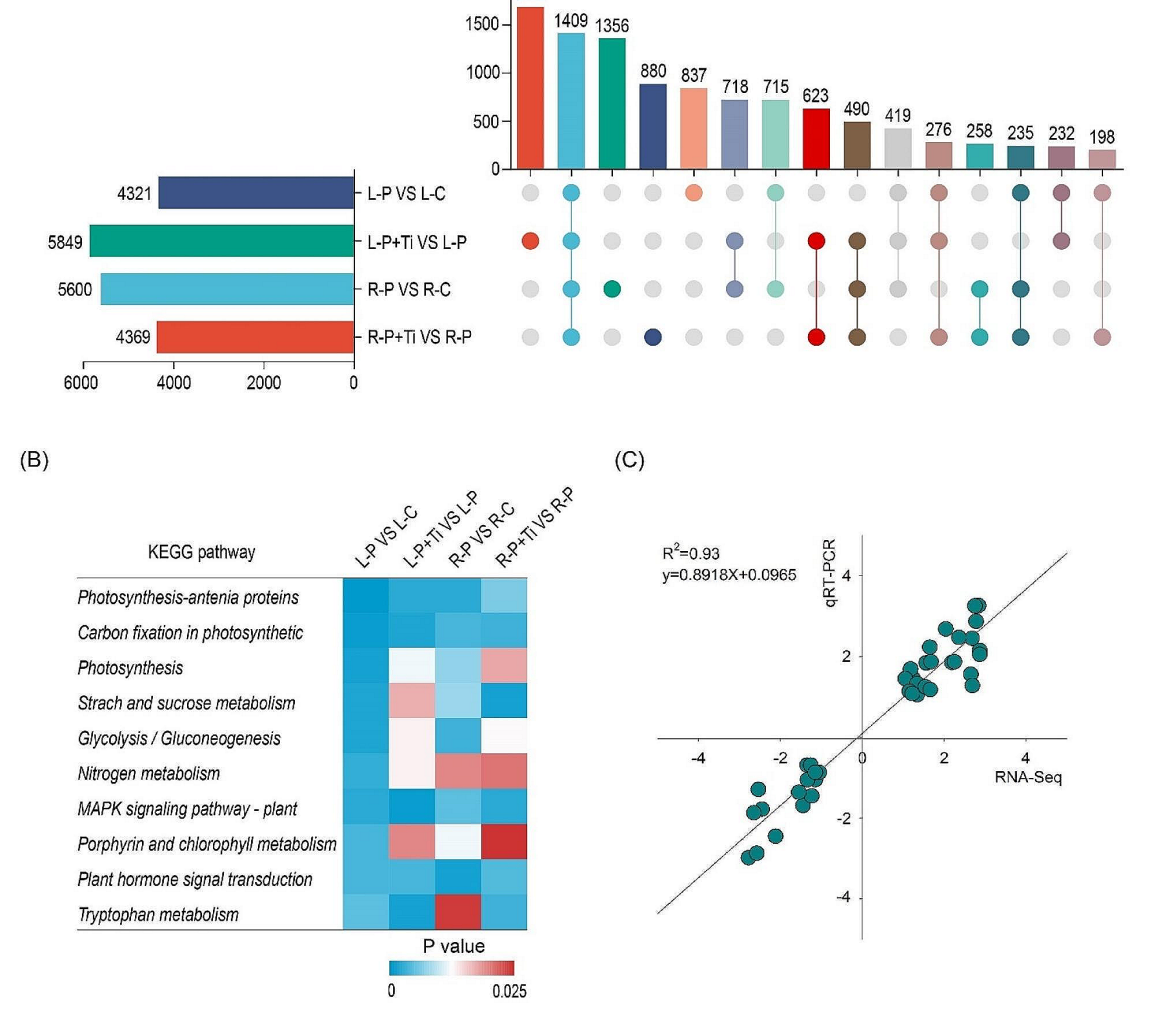
Nano-TiO_2_ and PSNPs induce transcriptome reprogramming in maize plants. (**A**) The overlap of DEGs between different groups. (**B**) KEGG enrichment analysis. Color scale is shown as significance level. (**C**) Validation of transcriptome data by qRT-PCR. R/L-C, control roots/leaves; R/L-P, PSNPs-treated roots/leaves; R/L-P + Ti, PSNPs + Nano-TiO_2_-treated roots/leaves

To enhance the understanding of the potential mechanism by which Nano-TiO_2_ mitigate PSNPs toxicity, transcriptome sequencing was conducted on the leaves and roots subjected to Nano-TiO_2_ treatment. In the comparisons of P vs. C and P + Ti vs. P, we identified 5,600 and 4,369 DEGs (|log_2_fold change| > 1) in the roots, and 4,321 and 5,849 DEGs in the leaves, respectively (Fig. 2A). Additional KEGG enrichment analysis of the DEGs revealed a substantial enrichment of these genes in pathways associated with photosynthesis, starch and sucrose metabolism, tryptophan metabolism, nitrogen metabolism, carbon fixation, and glycolysis (Fig. 2B). To validate the reliability of the transcriptome sequencing data, a random selection of eight candidate genes was subjected to qRT-PCR analysis. The results demonstrated a strong correlation between the RNA-Seq and qRT-PCR data for these genes, thereby confirming the accuracy of the transcriptome data (Fig. 2C).

### Nano-TiO_2_ modulates C metabolism

**Fig. 3:**
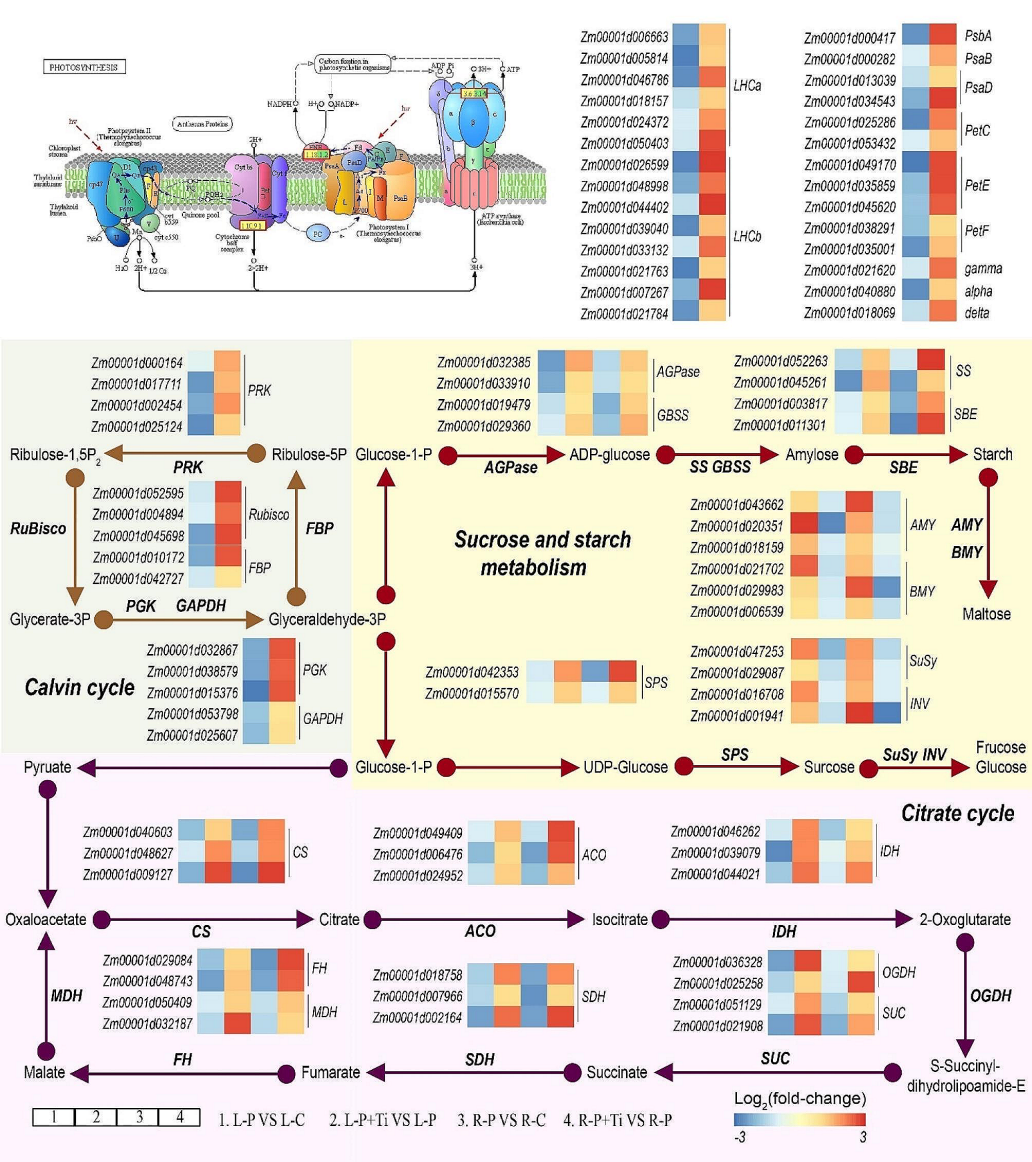
Effects of Nano-TiO_2_ on the transcription levels of genes involved in carbon metabolism. Color scale denotes log_2_(fold-change). *LHCa/LHCb*: *PS-I/PS-II light harvesting complex*; *PsbA*: *PS-II reaction center protein*; *PsbB/PsbD*: *PS-I reaction center protein*; *PetC*: *Iron-sulfur protein*; *PetE/PetF*: *Plastocyanin; gamma/alpha/delta: ATP synthase subunit; Rubisco*: *Ribulose bisphosphate carboxylase*; *PGK*: *Phosphoglycerate kinase*; *GAPDH*: *Glyceraldehyde phosphate dehydrogenase*; *FBP*: *Fructose-1,6-bisphosphatase*; *PRK*: *Phosphoribulokinase*; *AGPase*: *ADP Glucose pyrophosphorylase*; *SS*: *Starch synthase*; *GBSS*: *Granule-bound starch synthase*; *SBE*: *Starch branching enzyme*; *AMY*: *Alpha-amylase*; *BMY*: *Beta-amylase*; *SPS*: *Sucrose phosphate synthase*; *SuSy*: *Sucrose synthase*; *INV*: *Invertase*; *CS*: *Citrate synthase*; *ACO*: *Aconitase*; *IDH*: *Isocitrate dehydrogenase*; *OGDH*: *Oxoglutarte dehydrogenase*; *SUC*: *Succinyl-CoA synthetase*; *SDH*: *Succinate dehydrogenase*; *FH*: *Fumarate hydratase*; *MDH*: *Malate dehydrogenase*

**Fig. 4:**
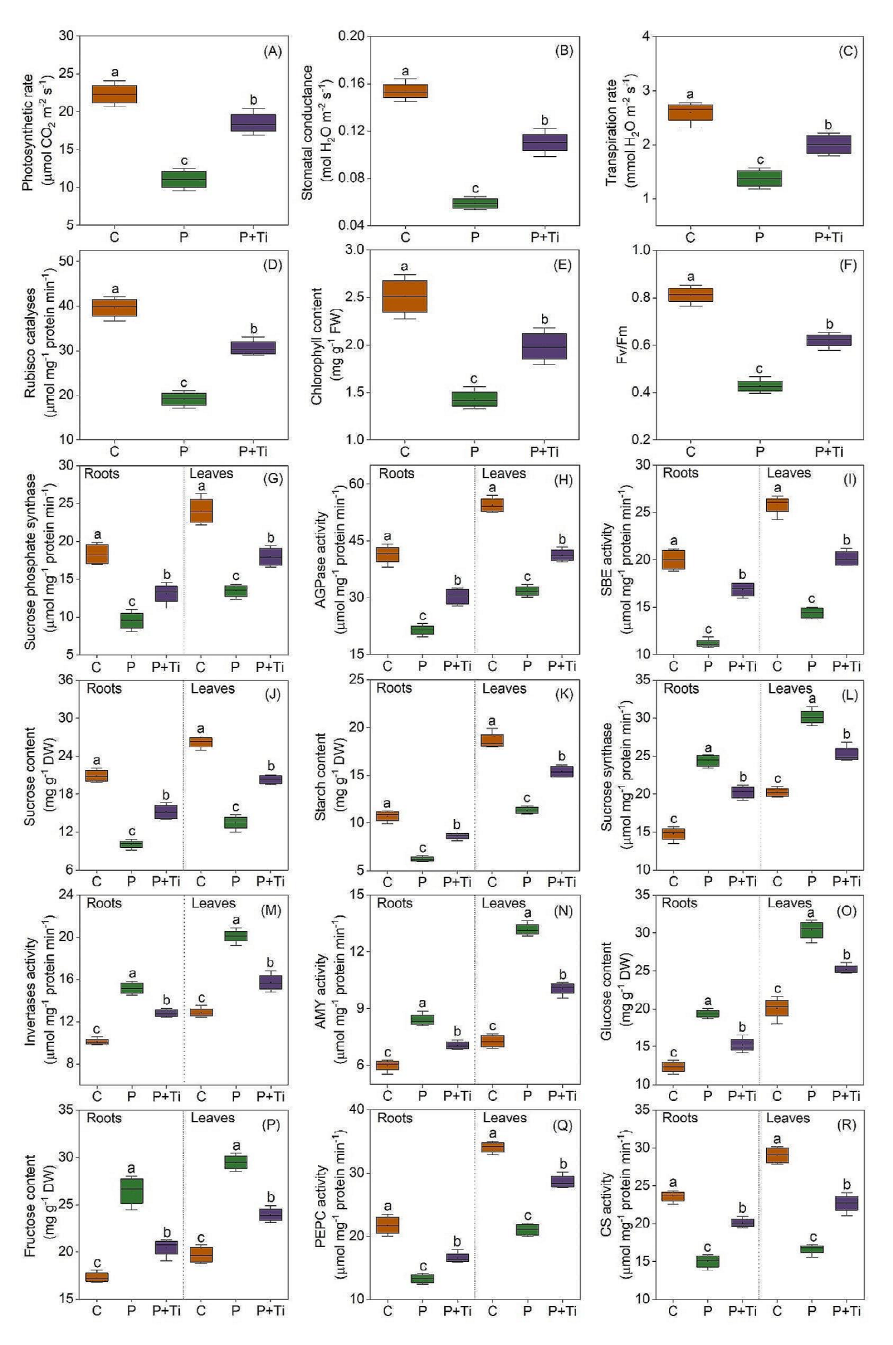
Effects of Nano-TiO_2_ on carbon metabolism in PSNPs-stressed maize plants. (**A**) photosynthetic rate; (**B**) stomatal conductance; (**C**) transpiration rate; (**D**) rubisco catalyses; (**E**) chlorophyll content; (**F**) Fv/Fm; (**G**) sucrose phosphate synthase (SPS) activity; (**H**) ADP glucose pyrophosphorylase (AGPase) activity; (**I**) starch branching enzyme (SBE) activity; (**J**) sucrose content; (**K**) starch content; (**L**) sucrose synthase (SuSy) activity; (**M**) invertases (INV) activity; (**N**) amylase (AMY) activity; (**O**) glucose content; (**P**) fructose content; (**Q**) phosphoenolpyruvate carboxylase (PEPC) activity; (**R**) citrate synthase (CS) activity. C: Control; Ti: Nano-TiO_2_; P + Ti: PSNPs + Nano-TiO_2_. Different letters indicate statistically significant differences (*n* = 4, *P* < 0.05)

The transcription of genes related to photosynthetic processes, such as the light harvesting complex (*LHC*), photosynthetic proteins (*Psb* and *Psa*), and electron transporters (*Pet* and *ATPase*), was significantly enhanced by Nano-TiO_2_ (Fig. 3). Furthermore, the application of Nano-TiO_2_ led to a notable increase in the photosynthetic rate (Fig. 4A). Moreover, there was a significant upregulation of genes associated with the Calvin cycle, including *Rubisco*, *glyceraldehyde phosphate dehydrogenase (GAPDH)*, *Phosphoglycerate kinase (PGK)*, *Fructose-1,6-bisphosphatase (FBP)*, and *Phosphoribulokinase (PRK)* (Fig. 4C). As a result, the rubisco activity in the leaves treated with Nano-TiO_2_ exhibited a significantly higher value compared to that of untreated plants (Fig. 4D). Moreover, the application of Nano-TiO_2_ demonstrated a significant enhancement in chlorophyll content by 38.6% and the Fv/Fm ratio by 44.8% (Fig. 4E and F).

**Fig. 5:**
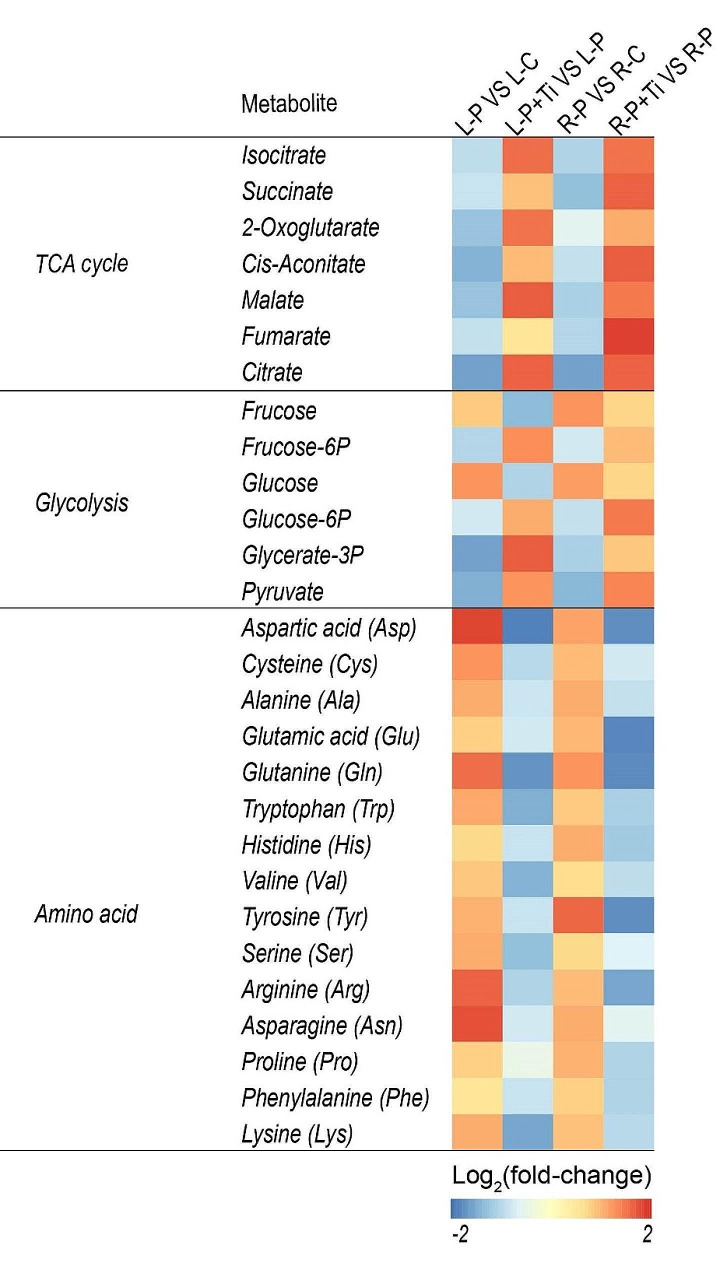
Effects of Nano-TiO_2_ on the contents of metabolites associated with carbon/nitrogen metabolism. Color scale denotes log_2_(fold-change)

Under PSNPs stress, the expression levels of genes involved in the biosynthesis of starch and sucrose, such as *AGP*, *GBSS*, *SS*, *SBE*, and *SPS*, were found to be upregulated in both the L-P + Ti/L-P and R-P + Ti/R-P comparisons (Fig. 3). Furthermore, the presence of Nano-TiO_2_ significantly enhanced the activities of SPS, AGPase, and SBE, as well as the levels of sucrose and starch (Fig. 4G-K). Moreover, Nano-TiO_2_ were observed to significantly reduce the transcription of genes associated with the degradation of starch and sucrose, including *AMY*, *BMY*, *SuSy*, and *INV* (Fig. 3). Consequently, the presence of Nano-TiO_2_ in plants led to a decrease in the activities of AMY, SuSy, and INV, as well as the levels of fructose and glucose, when compared to plants that were not treated (Fig. 4L-P). Moreover, when plants were exposed to PSNPs stress, the transcription of genes related to the tricarboxylic acid cycle (TCA), such as *CS, aconitase (ACO), isocitrate dehydrogenase (IDH), oxoglutarte dehydrogenase (OGDH), succinyl-CoA synthetase (SUC), succinate dehydrogenase (SDH), fumarate hydratase (FH), and malate dehydrogenase (MDH)*, exhibited a significant increase in response to the application of Nano-TiO_2_ (Fig. 3). Similarly, the utilization of Nano-TiO_2_ resulted in a notable enhancement in the activity of enzymes (including CS and PEPC) and the accumulation of metabolites associated with the TCA in maize plants (Figs. 4Q and R and 5). These findings demonstrate that Nano-TiO_2_ have the potential to enhance the plants’ capacity for sucrose synthesis.

### Nano-TiO_2_ improves N metabolism

**Fig. 6:**
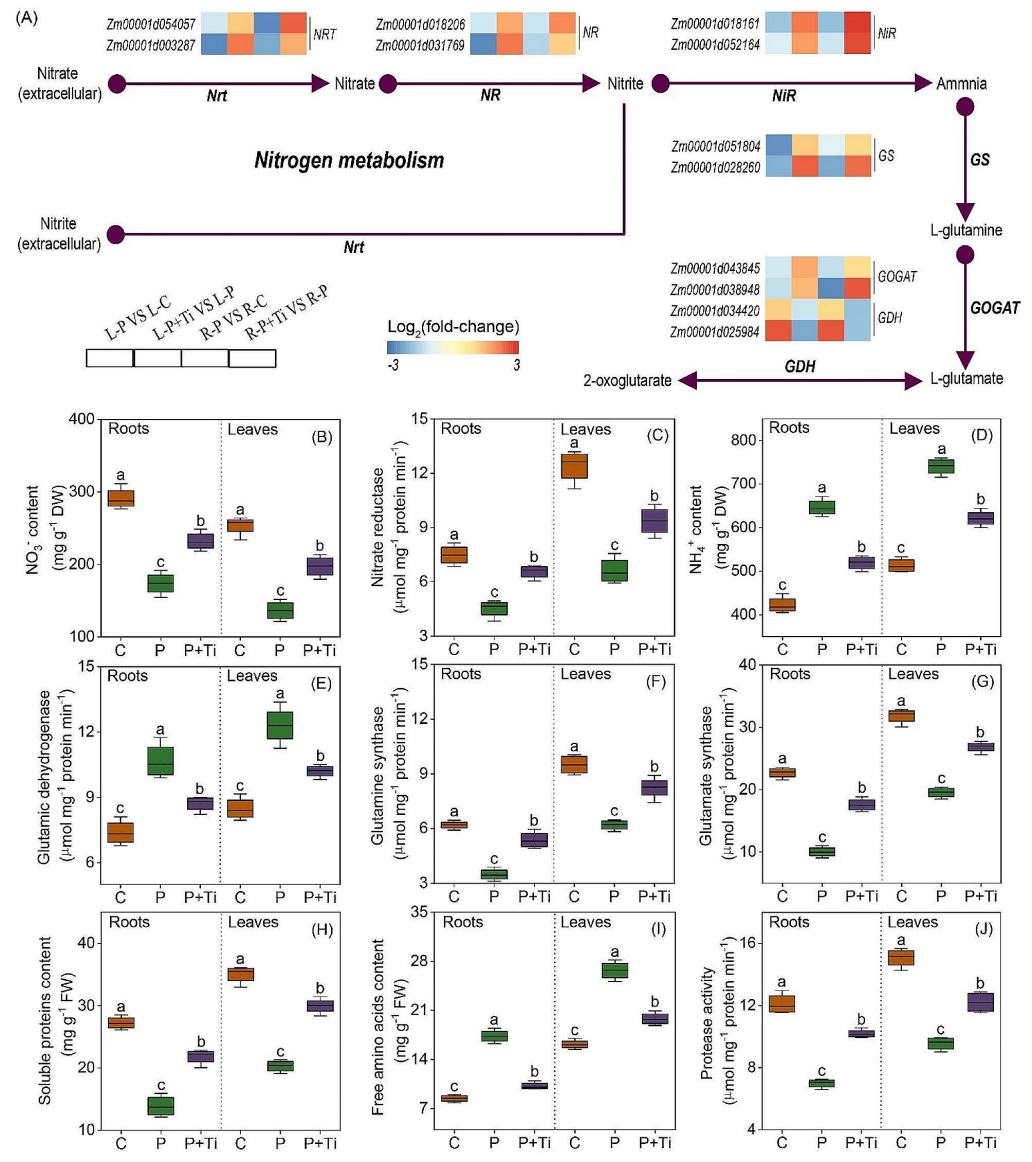
Effects of Nano-TiO_2_ on nitrogen metabolism in PSNPs-stressed maize plants. (**A**) the transcription levels of genes associated with nitrogen metabolism. Color scale denotes log_2_(fold-change). *NRT: Nitrate transporter; NR: Nitrate reductase; GS: Glutamine synthetase; GOGAT: Glutamate synthetase; GDH: Glutamate dehydrogenase.* Nano-TiO_2_ effects on nitrate (NO_3_^−^) content (**B**), NR activity (**C**), ammonia (NH_4_^+^) content (**D**), GDH activity (**E**), GS activity (**F**), GOGAT activity (**G**), soluble protein content (**H**), free amino acid content (**I**), and protease activity (**J**). C: Control; Ti: Nano-TiO_2_; P + Ti: PSNPs + Nano-TiO_2_. Different letters indicate statistically significant differences (*n* = 4, *P* < 0.05)

In light of PSNPs stress, the expression levels of genes related to nitrogen assimilation, specifically *NR* and *nitrate transporter (NRT)*, were observed to be upregulated by Nano-TiO_2_ (Fig. 6A). Consequently, the administration of Nano-TiO_2_ resulted in a notable augmentation of NR activity and NO_3_^−^ concentration (Fig. 6B and C). In the presence of PSNPs stress, the genes associated with GDH synthesis exhibited a significant upregulation in response to Nano-TiO_2_ (Fig. 6A). Nevertheless, the GDH activity and NH_4_^+^ content in both leaves and roots were found to be elevated by Nano-TiO_2_. Additionally, the utilization of Nano-TiO_2_ yielded a notable increase in the activities of GS and GOGAT by 32.8% and 37.5% in the leaves, and 54.5% and 75.3% in the roots, respectively (Fig. 6C-F).

During exposure to PSNPs stress, the presence of Nano-TiO_2_ significantly upregulated the transcription of genes associated with the biosynthesis of ribosomal proteins, as depicted in Fig. 4G. Furthermore, plants treated with Nano-TiO_2_ exhibited reduced protease activity and increased protein content. Moreover, the application of Nano-TiO_2_ resulted in a substantial decrease of 25.7% and 41.0% in the free amino acid content of both leaves and roots of maize plants, compared to untreated plants (Fig. 6G-I). In summary, the utilization of Nano-TiO_2_ can effectively enhance the nitrogen assimilation and protein synthesis capabilities of maize seedlings.

### Nano-TiO_2_ alleviates PSNPs-induced oxidative damage

**Fig. 7:**
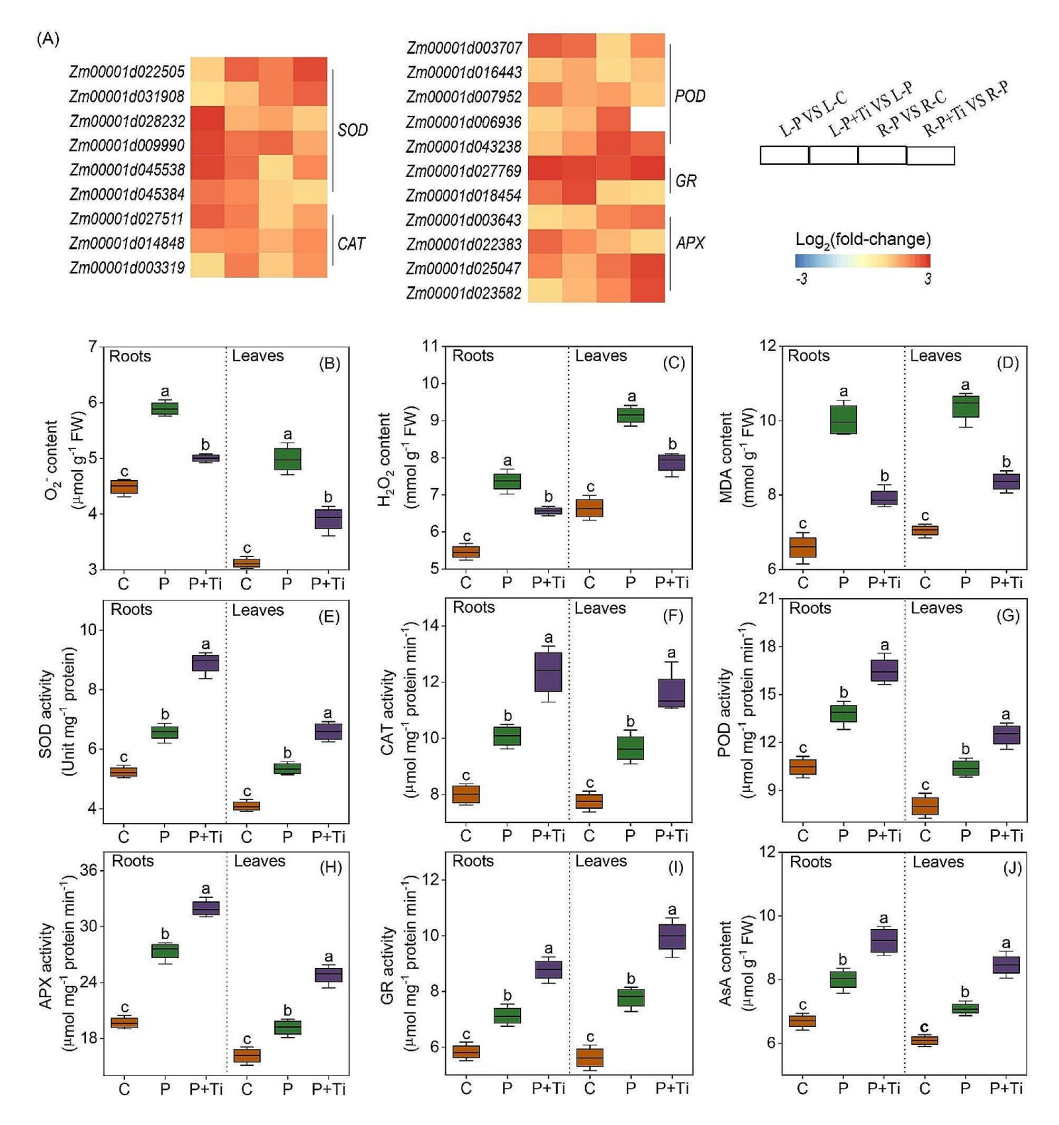
Effects of Nano-TiO_2_ on antioxidant capacity in PSNPs-stressed maize plants. (**A**) the transcription levels of genes involved in the biosynthesis of antioxidant enzyme. Color scale denotes log_2_(fold-change). *SOD*: *Superoxide dismutase*; *CAT*: *Catalase*; *POD*: *Peroxidase*; *APX*: *Ascorbate peroxidase*; *GR*: *Glutathione reductase*. Nano-TiO_2_ effects on superoxide anion radical (O_2_^•−^) content (**B**), hydrogen peroxide (H_2_O_2_) content (**C**), malondialdehyde (MDA) content (**D**), SOD activity (**E**), CAT activity (**F**), POD activity (**G**), APX activity (**H**), GR activity (**I**), and ascorbate (AsA) content (**J**). C: Control; Ti: Nano-TiO_2_; P + Ti: PSNPs + Nano-TiO_2_. Different letters indicate statistically significant differences (*n* = 4, *P* < 0.05)

During exposure to PSNPs stress, treatment with Nano-TiO_2_ result in a significant reduction in the levels of O_2_^•−^ by 18.3% and 16.7% in the leaves and roots, respectively (Fig. 7B). Additionally, the application of Nano-TiO_2_ resulted in a substantial decrease in the levels of H_2_O_2_ and MDA by 13.8% and 19.4% in the leaves, and 10.8% and 21.0% in the roots, respectively (Fig. 7C and D). Furthermore, under PSNPs stress conditions, there was a notable upregulation of genes associated with the synthesis of antioxidant enzymes, including *SOD*, *CAT*, *POD*, *APX*, and *GR* (Fig. 7A). Moreover, the enzymatic activities of antioxidant enzymes in plants treated with Nano-TiO_2_ exhibited a notable increase when compared to plants that were not subjected to such treatment (Fig. 7D-H). Furthermore, the concentration of AsA in maize plants treated with Nano-TiO_2_ demonstrated a significant increase in comparison to non-treated plants (Fig. 7I). These findings provide evidence to support the notion that Nano-TiO_2_ possess the ability to augment the antioxidant capacity of plants and shield their cell membranes from oxidative harm induced by PSNPs toxicity.

### Melatonin mediates Nano-TiO_2_ induced tolerance to PSNPs stress in maize plants

**Fig. 8:**
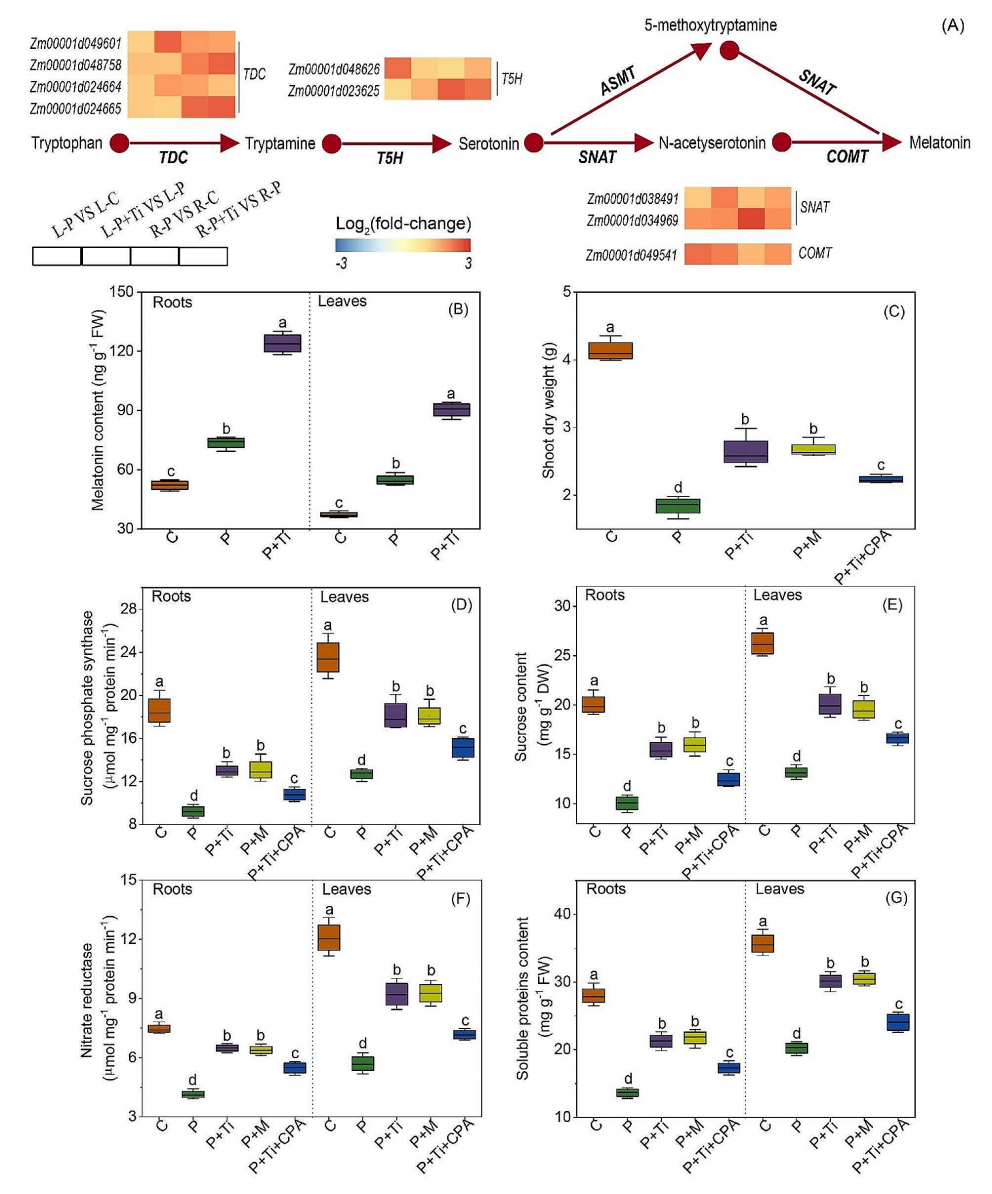
Melatonin mediates Nano-TiO_2_ induced tolerance to PSNPs stress. (**A**) the transcription levels of genes associated with melatonin synthesis. Scale bar denotes log_2_(fold-change). *TDC*: *tryptophan decarboxylase*; *T5H*: *tryptamine 5-hydroxylase*; *SNAT*: *serotonin N-acetyltransferase*; *COMT*: *caffeic-O-methyltransferase*. Effects of Nano-TiO_2_, melatonin and *P*-chlorophenylalanine (*p*-CPA, a melatonin synthesis inhibitor) on melatonin content (**B**), shoot dry weight (**C**), sucrose phosphate synthase (SPS) activity (**D**), sucrose content (**E**), nitrate reductase (NR) activity (**F**), soluble protein content (**G**). C: Control; Ti: Nano-TiO_2_; P + Ti: PSNPs + Nano-TiO_2_; P + M: PSNPs + melatonin; P + Ti + CPA: PSNPs + Nano-TiO_2_ + *p*-CPA; Different letters indicate statistically significant differences (*n* = 4, *P* < 0.05)

Under PSNPs stress, genes involved in the melatonin biosynthesis, including *Tryptophan decarboxylase (TDC)*, *Tryptamine 5-hydroxylase (T5H)*, *Serotonin N-acetyltransferase (SNAT)*, and *Caffeic-O-methyltransferase* (*COMT)*, were markedly upregulated by Nano-TiO_2_ (Fig. 8A). Similarly, treatment with Nano-TiO_2_ resulted in a significant increase the contents of endogenous melatonin in the leaves and roots (Fig. 8B).

In order to confirm the role of melatonin in Nano-TiO_2_ induced tolerance to PSNPs stress, we conducted experiments to examine the impact of exogenous melatonin and *p*-CPA, an inhibitor of melatonin synthesis, on the growth of maize plants. The results showed that the application of exogenous melatonin or Nano-TiO_2_ led to a significant promotion of plant growth under PSNPs stress conditions. However, the introduction of *p*-CPA diminished this promoting effect (Fig. 8C). In a similar vein, the exogenous administration of Nano-TiO_2_ or melatonin has been observed to substantially enhance the enzymatic activity associated with C and N metabolism, as well as the concentration of metabolites. Conversely, the utilization of *p*-CPA has been found to diminish these aforementioned effects (Fig. 8).

## Discussion

NPs can have negative effects on the physical characteristics, development, and photosynthetic processes of plants [58]. Our research has demonstrated that the introduction of exogenous Nano-TiO_2_ can mitigate the growth inhibitory effects caused by PSNPs (Fig. 1). This finding is consistent with previous studies that have shown the ability of Nano-TiO_2_ to enhance plant growth in the presence of environmental stressors, as evidenced by experiments conducted on stevia [15]. The process of plant growth involves the accumulation of biomass, which is primarily influenced by the intricate interplay of diverse metabolic pathways [21, 37]. Consequently, this investigation directs our research towards elucidating the correlation between metabolic processes and the facilitation of plant growth. The comprehensive analysis of the transcriptome, metabolome, and physiology will significantly contribute to a more profound comprehension of the role played by Nano-TiO_2_ in the regulation of growth-related metabolic processes.

Previous research has demonstrated that the utilization of Nano-TiO_2_ can enhance the photosynthetic efficacy of plants when subjected to salt stress [59]. Our investigation has revealed that the presence of Nano-TiO_2_ notably upregulated the expression of genes associated with the photosynthetic process, including *LHC*, *Psb*, and *Pet* (Fig. 3). These findings indicate that Nano-TiO_2_ have the potential to facilitate the absorption, transfer, and conversion of light energy within leaves when exposed to PSNPs stress. It is commonly observed that abiotic stress conditions typically lead to a decline in electron transport activity and photochemical efficiency, while concurrently promoting thermal energy dissipation [60]. In this particular instance, the utilization of electrons for ATP and NADPH synthesis is hindered, resulting in a substantial buildup of ROS [61, 62]. Our investigation has revealed that the administration of Nano-TiO_2_ can enhance the expression levels of genes linked to electron transport. These findings indicate that Nano-TiO_2_ play a significant role in facilitating electron transport and mitigating ROS generation. Furthermore, prior research has substantiated the capacity of Nano-TiO_2_ to effectively eliminate ROS [53]. Thereby, our results providing a more comprehensive understanding of the mechanism by which Nano-TiO_2_ enhance photosynthetic efficiency.

Photosynthesis serves as the fundamental process underlying the development of plant biomass and productivity, with carbohydrates produced through photosynthesis accounting for approximately 95% of plants’ dry matter. Our investigation revealed a noteworthy enhancement in SPS activity and sucrose levels in maize plants upon the application of Nano-TiO_2_. Moreover, the presence of Nano-TiO_2_ resulted in significantly elevated sucrose and starch concentrations in the roots of treated plants compared to those without Nano-TiO_2_, indicating a potential role of Nano-TiO_2_ in facilitating assimilate transportation to the roots (Fig. 4). The introduction of Nano-TiO_2_ resulted in a notable augmentation in the activity of TCA-related enzymes, thereby facilitating an increased availability of carbon skeleton and energy for the synthesis of amino acids (Fig. 4K and L). Consequently, it can be concluded that the utilization of Nano-TiO_2_ has the potential to enhance the assimilation of carbon and the synthesis of sucrose in maize plants.

Abiotic stress, particularly NPs stress, frequently diminishes the functionality of enzymes associated with nitrogen assimilation and impedes the process of nitrogen assimilation [63]. Our investigation has revealed that the introduction of Nano-TiO_2_ externally can notably augment the NO_3_^−^ content and bolster the activity of NR (Fig. 6A and B). Despite the decline in NR activity, there was an elevation in NH_4_^+^ concentration (Fig. 6C). The excessive presence of NH_4_^+^ poses toxicity to cells, likely due to the accumulation of NH_4_^+^ leading to disruptions in cytoplasmic pH and protein compression [18]. The assimilation of NH_4_^+^ into cells primarily occurs through the GS/GOGAT and GDH pathways. The GDH pathway exhibits limited affinity for NH_4_^+^ and is exclusively activated in the presence of inhibition of the GS/GOGAT pathway [64]. Our investigation revealed a substantial decrease in the activities of GS and GOGAT in maize plants subjected to PSNPs stress, potentially attributable to NH_4_^+^ accumulation. However, the external administration of Nano-TiO_2_ notably augmented the activities of GS and GOGAT while diminishing the activity of GDH, thereby facilitating the enhanced assimilation of NH_4_^+^ (Fig. 6D-F). Prior research has demonstrated that Nano-TiO_2_ can heighten the activities of GS and GOGAT in rice plants experiencing stress conditions [45].

The present study observed a significant decrease in protein content in both maize leaves and roots under PSNPs stress, accompanied by a significant increase in free amino acid content (Fig. 6H and I). This finding aligns with previous research, which also reported a correlation between protein degradation in stressed plants and the accumulation of free amino acids [50, 51]. It is worth noting that the majority of soluble proteins in plants function as enzymes involved in diverse metabolic pathways. Consequently, the analysis and quantification of soluble protein content serve as a crucial indicator for assessing plant metabolism [42]. For instance, the presence of aluminum toxicity leads to the degradation of chloroplast proteins, accumulation of free amino acids, and diminished function of plastid enzymes such as GS [16]. Our investigation revealed that the application of Nano-TiO_2_ resulted in an increase in soluble protein content in maize plants, maintained stable chloroplast function, and enhanced nitrogen assimilation ability (Fig. 7). Shiri’s research also corroborated that Nano-TiO_2_ significantly augmented the capacity for protein synthesis in leaves subjected to salt stress [59]. Collectively, our findings indicate that Nano-TiO_2_ have the potential to stimulate protein synthesis in maize seedlings experiencing PSNPs stress.

Under normal environmental circumstances, the equilibrium between the generation and elimination of ROS within plant cells remains dynamic. However, when plants are subjected to stress stimuli, this equilibrium is disrupted, leading to a substantial buildup of ROS and disturbances in metabolic processes [52]. Recent investigations have substantiated the ability of Nano-TiO_2_ to safeguard cells against oxidative stress triggered by environmental factors [15, 23]. This particular study has ascertained that Nano-TiO_2_ possess the capability to significantly diminish the levels of O_2_^•−^, H_2_O_2_, and MDA in both maize leaves and roots (Fig. 7B-D). Consequently, these findings propose that Nano-TiO_2_ exhibit the potential to alleviate oxidative damage induced by PSNPs toxicity.

Plants have developed comprehensive defense mechanisms, consisting of enzymatic antioxidant systems (e.g., SOD, CAT, POD) and non-enzymatic antioxidant systems (e.g., AsA, GSH), to safeguard cells against potential damage caused by ROS [54]. Numerous studies have substantiated the capacity of Nano-TiO_2_ to enhance the activity of antioxidant enzymes, particularly in the presence of environmental stress [53]. The findings of this study demonstrate that the external application of Nano-TiO_2_ can substantially enhance the activity of antioxidant enzymes in both maize leaves and roots (Fig. 7E-I). Furthermore, the AsA-GSH cycle serves as a crucial mechanism for plants to combat oxidative stress induced by heavy metal exposure [55]. Our investigation has demonstrated that the application of Nano-TiO_2_ can effectively elevate the levels of AsA in maize plants (Fig. 7J). Taken together, these findings provide evidence that Nano-TiO_2_ possess the ability to mitigate oxidative harm by augmenting the antioxidant potential of maize seedlings.

Melatonin, an indole amine, is ubiquitously present in organisms and plays a significant role in numerous physiological and biochemical processes in plants [25, 56]. Recent research has demonstrated that melatonin functions as a signaling molecule in plants, regulating growth and development, while also conferring resistance against diverse biological and abiotic stresses, thereby augmenting plant stress resilience [66, 67]. In the present study, it was observed that the utilization of Nano-TiO_2_ resulted in a noteworthy augmentation of endogenous melatonin levels (Fig. 8B), indicating a potential association between melatonin and the ameliorative impact of Nano-TiO_2_ on PSNPs toxicity. This hypothesis was further substantiated through inhibitor experiments (Fig. 8). In conclusion, our findings provide evidence that melatonin plays a role in the promotion of maize growth induced by Nano-TiO_2_, potentially through its regulation of C and N metabolism.

## Conclusions

The results of our study indicate that the application of Nano-TiO_2_ can effectively mitigate the inhibitory effects of PSNPs stress on maize growth. Specifically, under PSNPs stress conditions, the exogenous application of Nano-TiO_2_ significantly enhanced various physiological parameters, including photosynthetic rate, NO_3_^−^ content, sucrose content, and protein content in plants. This resulted in the maintenance of relative stability in C and N metabolism. Furthermore, Nano-TiO_2_ can alleviate oxidative damage through the activation of the antioxidant defense system. Moreover, Nano-TiO_2_ significantly elevated the content of endogenous melatonin in maize plants. Based on these findings, our findings indicate that Nano-TiO_2_ alleviates polystyrene PSNPs-induced growth inhibition in maize requires melatonin signaling. These discoveries offer novel perspectives for comprehending the mechanism by which Nano-TiO_2_ mitigates PSNPs toxicity, and providing valuable implications for the cultivation of maize.

### Electronic supplementary material

Below is the link to the electronic supplementary material.

Supplementary Material 1

## Data Availability

No datasets were generated or analysed during the current study.
