# Supplementary material for: Titanium dioxide nanoparticles alleviates polystyrene nanoplastics induced growth inhibition by modulating carbon and nitrogen metabolism via melatonin signaling in maize
Source: J Nanobiotechnology. 2024 May 17;22:262. doi: 10.1186/s12951-024-02537-x (PMC11100085; doi:10.1186/s12951-024-02537-x)
Supplement: Supplementary file 1 — Supplementary Material 1 [file 12951_2024_2537_MOESM1_ESM.docx]

**Table S1.** Gene specific primers used in this study.

| Transcript_ID | Annotation | Forward | Reverse |
| --- | --- | --- | --- |
| *Zm00001d052263* | *starch synthase* | CTTCACCTCCCAATCCTTTGA | GCGATGCCTATGCTTTCCA |
| *Zm00001d045261* | *starch synthase* | TTGTTAATGCCATCCAGATT | CCTTCCCAGGAGGACTTG |
| *Zm00001d001941* | *invertase* | CGACAAGTTCCGGGACCCTT | TCGTCACCCTCCTCCTTCACC |
| *Zm00001d043662* | *amylase* | GTCGTTGTTGGGTGTAATG | TGAAATCCCTGCCCTTAT |
| *Zm00001d009127* | *citrate synthase* | CTCACGCCATACGAGTTT | CGCTTATCCCTGTTGTCTC |
| *Zm00001d049409* | *aconitase* | GCGGCAGTTCTCGTGATT | GATTTCCCATTGTCGGTAG |
| *Zm00001d018206* | *nitrate reductase* | ATCCTCGCCTACATGCAGAACG | TCCGCTTGAGCCACTTGACC |
| *Zm00001d031769* | *nitrate reductase* | CGGCTACACCATGAAAGGATACGC | GGACCAGAAGCACCAGCACCAG |
| *Zm00001d028232* | *superoxide dismutase* | TAGCCAATGAAGATGGAGATG | CCCAGAATGGAATGAGGC |
| *Zm00001d009990* | *superoxide dismutase* | GAAGCACCACGCCACCTA | CCAGCCAAGTTTCCCATG |
| *NM_001329666.1* | *Polyubiquitin* | TGGTTGTGGCTTCGTTGGTT | GCTGCAGAAGAGTTTTGGGTACA |
